# Intestinal Epithelial Stem/Progenitor Cells Are Controlled by Mucosal Afferent Nerves

**DOI:** 10.1371/journal.pone.0016295

**Published:** 2011-02-09

**Authors:** Ove Lundgren, Mats Jodal, Madeleine Jansson, Anders T. Ryberg, Lennart Svensson

**Affiliations:** 1 Section of Physiology, Sahlgrenska Academy, University of Gothenburg, Göteborg, Sweden; 2 Section of Pharmacology, Institute of Neuroscience and Physiology, Sahlgrenska Academy, University of Gothenburg, Göteborg, Sweden; 3 Department of Molecular Virology, Linköping University, Linköping, Sweden; Emory University, United States of America

## Abstract

**Background:**

The maintenance of the intestinal epithelium is of great importance for the survival of the organism. A possible nervous control of epithelial cell renewal was studied in rats and mice.

**Methods:**

Mucosal afferent nerves were stimulated by exposing the intestinal mucosa to capsaicin (1.6 mM), which stimulates intestinal external axons. Epithelial cell renewal was investigated in the jejunum by measuring intestinal thymidine kinase (TK) activity, intestinal ^3^H-thymidine incorporation into DNA, and the number of crypt cells labeled with BrdU. The influence of the external gut innervation was minimized by severing the periarterial nerves.

**Principal Findings:**

Luminal capsaicin increased all the studied variables, an effect nervously mediated to judge from inhibitory effects on TK activity or ^3^H-thymidine incorporation into DNA by exposing the mucosa to lidocaine (a local anesthetic) or by giving four different neurotransmitter receptor antagonists i.v. (muscarinic, nicotinic, neurokinin1 (NK1) or calcitonin gene related peptide (CGRP) receptors). After degeneration of the intestinal external nerves capsaicin did not increase TK activity, suggesting the involvement of an axon reflex. Intra-arterial infusion of Substance P (SP) or CGRP increased intestinal TK activity, a response abolished by muscarinic receptor blockade. Immunohistochemistry suggested presence of M3 and M5 muscarinic receptors on the intestinal stem/progenitor cells. We propose that the stem/progenitor cells are controlled by cholinergic nerves, which, in turn, are influenced by mucosal afferent neuron(s) releasing acetylcholine and/or SP and/or CGRP. In mice lacking the capsaicin receptor, thymidine incorporation into DNA and number of crypt cells labeled with BrdU was lower than in wild type animals suggesting that nerves are important also in the absence of luminal capsaicin, a conclusion also supported by the observation that atropine lowered thymidine incorporation into DNA by 60% in control rat segments.

**Conclusion:**

Enteric nerves are of importance in maintaining the intestinal epithelial barrier.

## Introduction

The intestinal epithelium consists of a single layer of columnar cells about 25 µm high. It represents an outer surface of the organism exposed to luminal contents of widely varying composition. The epithelium is of great importance for the survival of the organism, since chemicals and/or microorganisms, if allowed to pass the epithelial barrier, may represent a deadly threat. Thus, the intestinal epithelium may be looked upon as being an important part of innate immunity. The maintenance of the epithelium is secured by a rapid renewal, the epithelium being replaced every 3–5 days in mammals [1]. The key cells in this event are the intestinal stem and progenitor cells located in the intestinal crypts. The present study describes animal experiments, which suggest that there is a nervous reflex control of the stem/progenitor cells and, hence, rate of epithelial cell renewal.

The nervous control of the gastrointestinal tract is exerted by two systems: the extrinsic, efferent sympathetic and parasympathetic nerves and the intrinsic enteric nervous system (ENS). The latter represents a nervous system in the gastrointestinal tract, controlling epithelial transport, motility and blood flow [2]. ENS is composed of two major nerve plexuses, the myenteric and the submucosal and of their interconnections. Most of the neurons of the ENS are confined to the gastrointestinal wall, but extrinsic nerves are also found in the ENS. Two major types of nervous reflexes can be identified in the ENS: intramural reflexes and axon reflexes. Intramural reflexes are confined to the intestinal wall. The peristaltic reflex is an example of an intramural reflex initiated by luminal distension [2]. Branching external afferent nerves constitute the morphological bases of axon reflexes.

Most investigations of the nervous control of gastrointestinal functions have been concerned with its control of motility, blood flow or epithelial transport. Although the extrinsic control of epithelial cell proliferation usually is discussed in terms of growth factors, peptide hormones and inflammatory mediators (for review, see reference [3]), there are observations to suggest also a nervous control of intestinal cell renewal. In most of these studies the influence of the extrinsic nerves has been investigated, suggesting that both branches of the extrinsic innervation may increase mitotic rate in the crypt cells [4]–[6]. Chemical ablation of the myenteric plexus leads to an increased crypt cell proliferation both in the small intestine [6]–[8] and in the colon [9], [10], indicating that myenteric nerves may exert an inhibitory influence on intestinal cell renewal.

We have earlier demonstrated that ENS plays an important role in the control of fluid transport. In fact, in many, if not most, types of acute diarrhea, an activation of ENS is the major mechanism underlying the observed intestinal fluid loss [11]–[13]. Diarrhea can be looked upon as being an innate immunity response, since the secreted fluid dilutes the potentially noxious agent(s). The maintenance of the epithelial barrier is also important for innate immunity. Therefore, we decided to investigate if intestinal contents, via the ENS, also may influence epithelial cell renewal. Most experiments were performed on intestinal segments that had been acutely denervated by cutting the nerves accompanying the superior mesenteric artery, minimizing the influence of the external innervation. The ENS was activated in two different ways: In one type of experiments an intramural secretory reflex was stimulated by exposing the intestinal mucosa to cholera toxin [11]. In another series we utilized capsaicin, the “hot” agent of red pepper, to activate thin mucosal afferents [14]. Capsaicin is known to activate the TRPV1 receptor. Cell renewal was studied in three different ways: We determined deoxythymidine kinase (TK) activity in the intestinal wall. TK facilitates the phosphorylation of deoxythymidine, associated with the formation of DNA [15]. We also measured the rate of incorporation of radioactively labeled thymidine into intestinal DNA. Finally, epithelial cell renewal was estimated by means of bromodeoxyuridine (BrdU), a thymidine analogue possible to localize at the cellular level in tissue sections with immunohistochemistry.

## Results

### Rapid cell renewal in the intestine is located to the crypts

**Figure 1:**
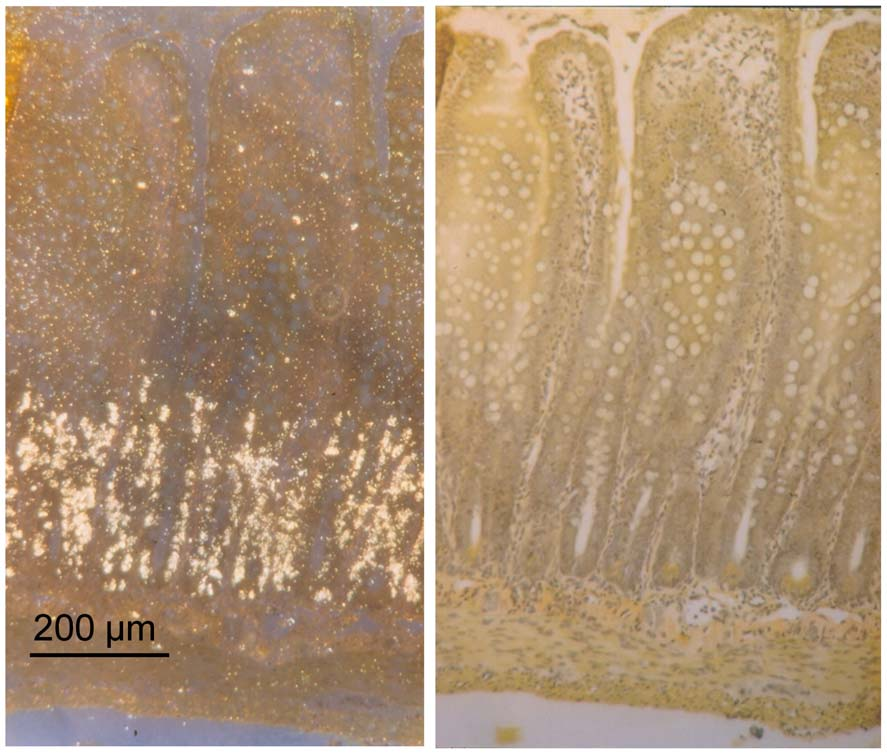
Autoradiography of rat small intestine after giving tritiated thymidine i.v. The labeled compound (50 µCi ^3^H-methyl-thymidine) was administred 6 h prior to removing the intestine The left panel illustrates dark field microscopy of an autoradiogram showing radiolabeled cells (white spots) almost exclusively located in the epithelial layer of the crypts of Lieberkühn. To the right the same section is seen in light microscopy. Van Gieson.

**Figure 2:**
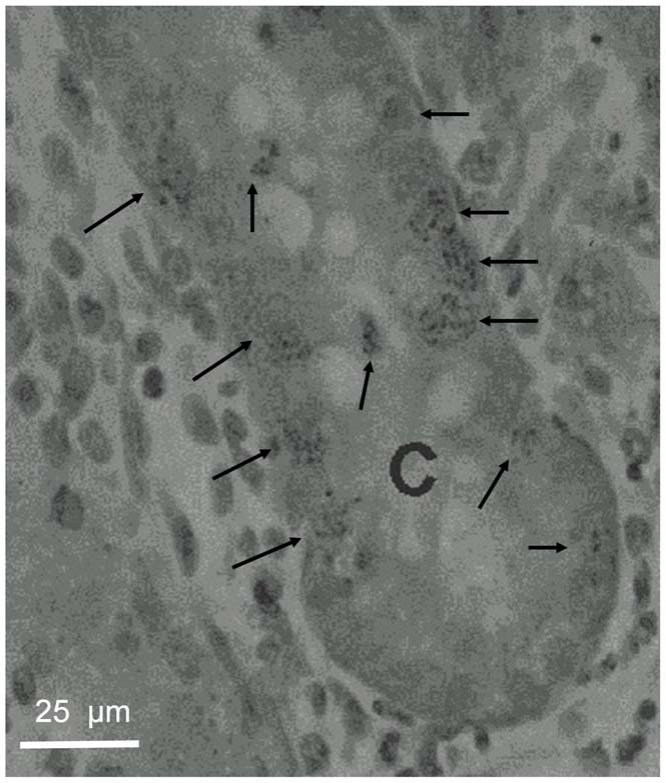
The distribution of ^3^H-methyl-thymidine in an intestinal crypt (C) as shown by autoradiography. The labeled compound (50 µCi ^3^H-methyl-thymidine) was administred 6 h prior to removing the intestine. Only cell nuclei were found to be labeled (arrows).

**Figure 3:**
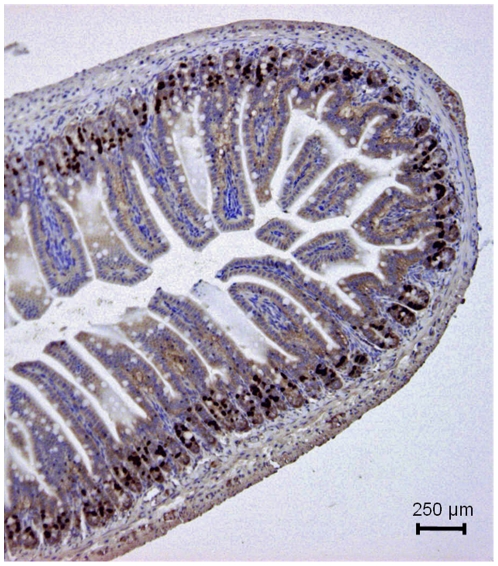
BrdU labeling of the small intestine after i.v. administration. BrdU (50 mg/kg b.wt.), a thymidine analogue, was given 2 h prior to removing the intestine. The dark labeling by BrdU is exclusively located to the nuclei of intestinal crypt cells. Mayer's haematoxylin.

TK activity and methyl-^3^H-thymidine incorporation into DNA was measured in the whole intestine. Hence, it was important to ensure that the recorded activity/incorporation mainly reflected events in the intestinal crypt stem/progenitor cells. This was tested in two ways. First, the left panel of Figure 1 shows an autoradiograph of the small intestine from a rat given methyl-^3^H-thymidine i.v. 6 h prior to the removal of the intestine. Dark field microscopy shows that the labeled thymidine was mainly located to the crypts, where intestinal epithelial stem/progenitor cells are found [16]–[18]. The right panel shows the same histological section in regular light microscopy. Figure 2 illustrates the localization of labeled thymidine at a higher magnification in intestinal crypts. The photographic grains are only seen above the nuclei of the cells (indicated by arrows). Second, in another type of experiments BrdU (a thymidine analogue) was given i.v. (50 mg kg^−1^). Two h after the i.v. injection, BrdU immunoreactivity was exclusively found in the crypt epithelial cells (Figure 3).

### Cholera toxin does not increase rate of epithelial renewal

Cholera toxin evokes an intestinal fluid secretion mainly by stimulating nervous reflex(es) contained within the ENS [11], [13]. Exposing the intestinal mucosa to cholera toxin (20 µg per mL) for three hours did not influence TK activity (91.2±11.2% (mean ± s.e.m) of control; n = 5; p = 0.5), but decreased the incorporation of labeled thymidine into DNA (84.4±5.5% of control; n = 5; p<0.05).We conclude that in the present short term experiments nervous reflexes activated by cholera toxin do not increase rate of epithelial renewal.

### Luminal capsaicin increases rate of epithelial renewal

**Figure 4:**
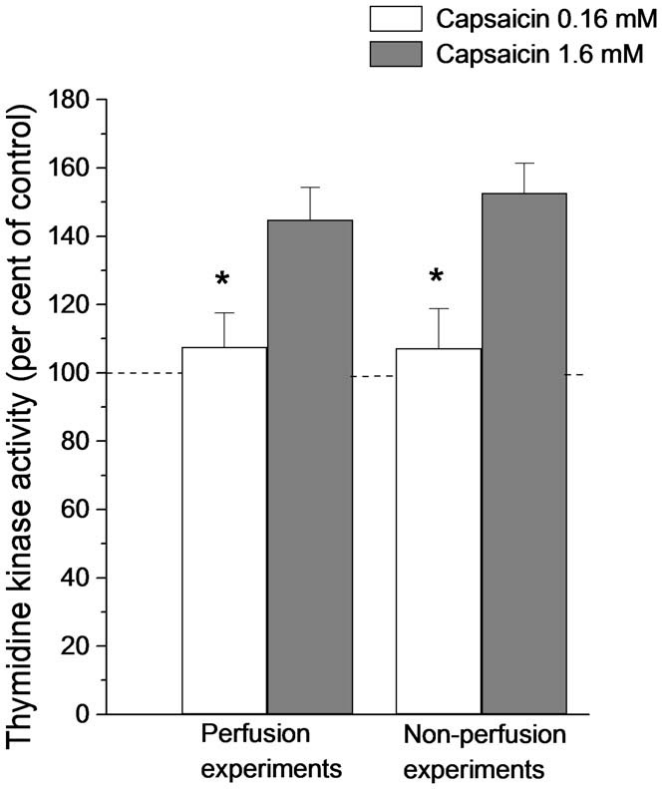
The effect on intestinal thymidine kinase activity of exposing the intestinal mucosa to capsaicin (0.16 or 1.6 mM). Thymidine kinase activity is expressed in per cent of activity measured in control segments (no capsaicin). The experiments were performed using two types of preparations. In most experiments only the proximal ends of the segments were cannulated with plastic tubing and the intestinal segment was perfused by means of a pump at a rate of 20 µL per min (“perfusion experiments”; n = 6). In other experiments the distal ends were also provided with plastic tubing closed by plugs (“non-perfusion experiments”; n = 7). Asterisks indicate statistical significance. Mean ± s.e.m.

Two experimental protocols were used in the capsaicin experiments, “non-perfusion experiments” and “perfusion experiments” (see Methods). Since both techniques indicated that a 1.6 mM capsaicin solution evoked a consistent increase of TK activity (152.7±8.6% of TK activity in control segments (non-perfusion experiments; n = 7; p-value <0.05) and 149.8±14.4% of control segments (perfusion experiments; n = 6; p-value <0.05; Figure 4), this concentration was used in subsequent experiments, most of which were performed with the perfusion technique.

**Figure 5:**
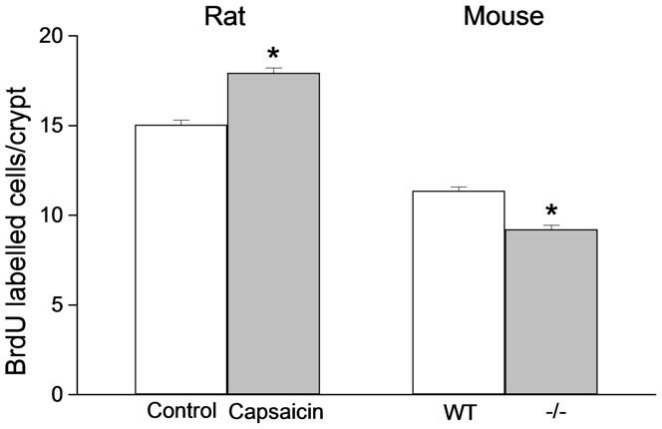
Rat: BrdU labeled cells per crypt in intestinal segments exposed to a capsaicin or a control solution. BrdU (50 mg/kg bwt) was given i.v. 2 h prior to removal of the intestinal segments. The number of labeled cells in 255 crypts in control (n = 5) and in 249 crypts in capsaicin segments (n = 5) were counted. Asterisk indicates statistical significance. Mean ± s.e.m. **Mouse: BrdU labeled cells per crypt in wild type or capsaicin receptor knock-out mice.** Five capsaicin receptor knock-out mice (−/−; 225 crypts) and in five wild type mice (WT; 215 crypts) were investigated. BrdU (100 mg/kg b.wt.) was administered i.p. 2 h before removal of the small intestine. Asterisk indicates statistical significance. Mean ± s.e.m.

The incorporation of labeled thymidine into DNA was significantly increased after 2 h capsaicin perfusion (178.0±30.1% of incorporation in control segments; n = 6; p<0.05). Furthermore, the number of BrdU labeled crypt cells increased concomitantly in the capsaicin group as shown on left part of Figure 5, based on counting labeled cells in 255 crypts in control and 249 crypts in capsaicin segments (control segments: 15.0±0.3 labeled cells/crypt; capsaicin segments: 17.9±0.3 labeled cells/crypt; n = 5; p<0.01). Hence, exposing the intestinal mucosa to 1.6 mM capsaicin increased TK activity and ^3^H-thymidine incorporation into DNA reflecting events in crypt epithelial cells as confirmed by the BrdU experiments.

### The capsaicin effect on epithelial renewal is mediated by nerves

Capsaicin was used to activate selectively thin afferent nerve fibers presumably via the vallinoid receptor 1 (TRPV1) [14]. We explored if capsaicin indeed activated afferent nerves by monitoring changes of heart rate on line after luminal administration of a 1.6 mM capsaicin. The periarterial nerves were not severed in these experiments. Within ten minutes 1.6 mM capsaicin heart rate increased by 46.2±8.2 min^−1^, whereas heart rate was not affected (−4.7±5.1 min^−1^) in control experiments (n = 6; p<0.05), suggesting that luminal capsaicin activates nervous afferents.

In one series of experiments the extrinsic intestinal nerves were allowed to degenerate for 2–3 weeks after cutting the nerves around the superior mesenteric artery. The success of the denervation procedure was tested by immunohistochemical determinations of tyrosine hydroxylase in the intestine, located to extrinsic adrenergic nerve fibers [19]. Absence of labeled nerve fibers was taken to indicate a successful denervation. Exposing such “chronically” denervated intestinal segments to capsaicin did not significantly increase TK activity (102.7±3.1% of control; n = 5). This is significantly lower than the capsaicin effect observed in “acutely” denervated segments (144.1±8.3%; n = 18; p<0.01).

**Figure 6:**
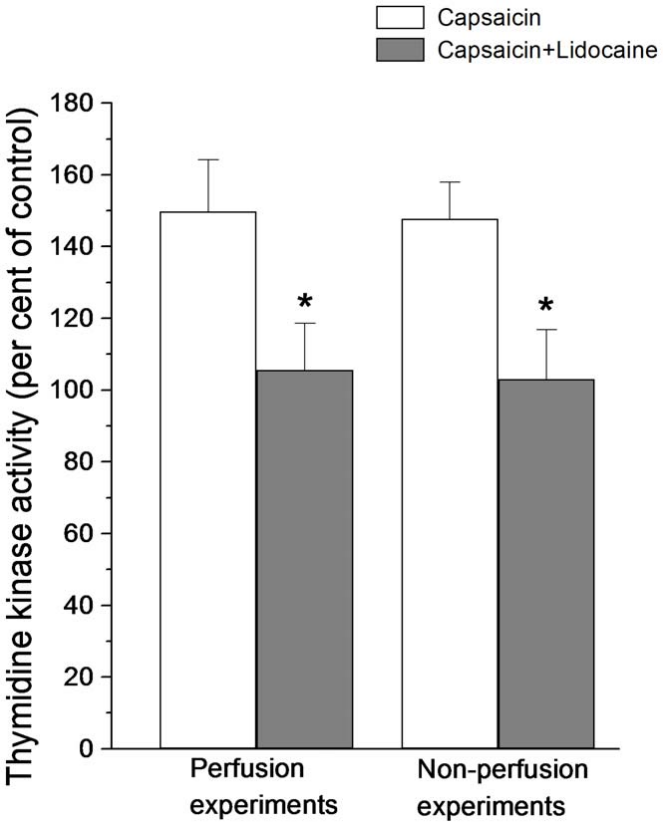
The effect of capsaicin on intestinal thymidine kinase activity after anesthetizing the mucosa with lidocaine. Thymidine kinase activity is expressed in per cent of activity measured in control segments (no capsaicin). Asterisks indicate statistical significance between the two groups of experiments (perfusion experiments, n = 8; non-perfusion experiments, n = 7). Mean ± s.e.m.

To further investigate the possible involvement of nerves in the capsaicin response, the intestinal mucosa was anesthetized with lidocaine (see Methods). In these experiments (Figure 6) no significant increase of TK activity was recorded (perfusion experiments: TK activity in capsaicin segments 97.5±14.4% of control; n = 8; p = 0.58; non-perfusion experiments: TK activity in capsaicin segments 103.1±13.7% of control; n = 7; p = 0.74), indicating a nervous involvement in the capsaicin response.

Indirect evidence for an involvement of nerves was also obtained in experiments with the TPRV1 receptor blocker capsazepine (3 mg/kg i.v.) given prior to the intraluminal administration of capsaicin. In these experiments the TK activity of the capsaicin segment was 97.2±7.3% (n = 7) of that measured in corresponding control segments (p<0.01 compared to a value of 144.1±8.3 per cent in experiments without capsazepine; n = 18). Hence, the capsaicin effect on TK activity was mediated by a TPRV1 receptor. Since the TPRV1 receptor is known to be located on many extrinsic afferent axons[14], the results support the proposal that the capsaicin effect involves nerves.

Finally, hexamethonium (a cholinergic, nicotinic receptor blocker; 10 mg/kg b.wt.; n = 8) significantly attenuated the effect of capsaicin on TK activity measuring 96.5±10.7% of the activity in capsaicin free segments (p<0.01 compared to 144.1±8.3 per cent in experiments without hexamethonium; n = 18). These results suggest that the capsaicin effect was mediated by ENS reflex(es) containing at least one cholinergic synapse.

### The capsaicin effect on epithelial renewal is mediated by muscarinic and peptidergic receptors

**Figure 7:**
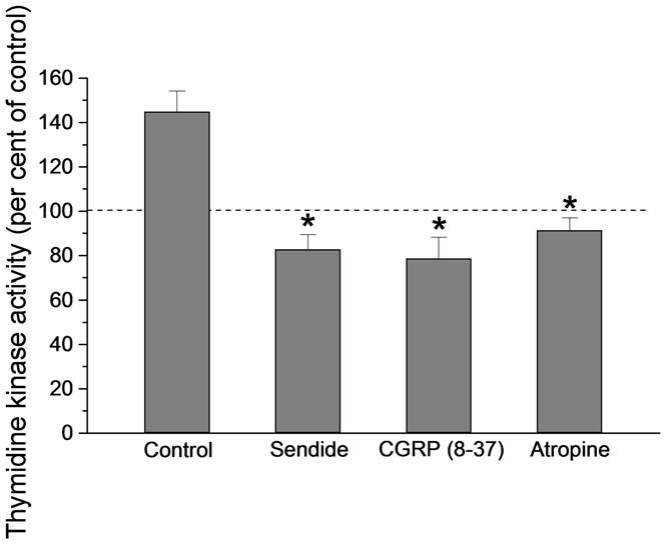
The effect of neurotransmitter receptor antagonists on capsaicin evoked increase of thymidine kinase activity. The receptor antagonists tested were: Sendide (NK1 receptor antagonist); 8–37 α-GGRP (CGRP receptor antagonist); atropine (muscarinic receptor antagonist). Thymidine kinase activity is expressed in per cent of activity measured in control segments (no capsaicin). N = 6 for each antagonist experiments. Asterisks indicate statistical significance compared to control. Mean ± s.e.m.

**Figure 8:**
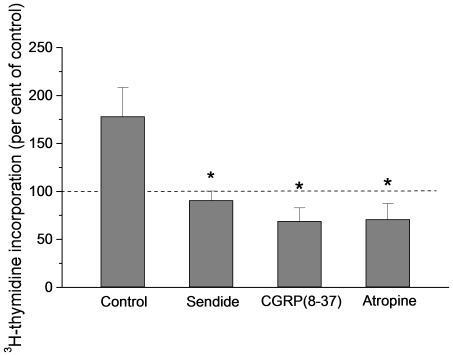
The effect of neurotransmitter receptor antagonists on capsaicin evoked increase of ^3^H-thymidine incorporation into DNA. The receptor antagonists tested were: Sendide (NK1 receptor antagonist); 8–37 α-GGRP (CGRP receptor antagonist); atropine (muscarinic receptor antagonist). The receptor blockers were given as described in Methods. ^3^H-thymidine incorporation is expressed in per cent of incorporation measured in control segments (no capsaicin). Asterisks indicate statistical significance compared to control. Mean ± s.e.m.

**Figure 9:**
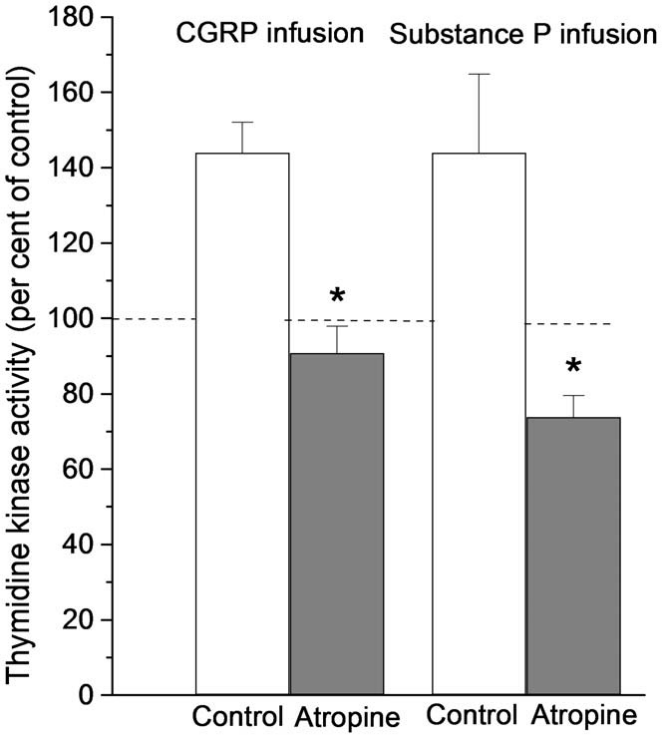
Thymidine kinase activity in intestinal segments after i.a. infusion of substance P or CGRP. The peptides were infused for one hour at a rate of 10−9 mol per minute with or without prior administration of atropine (0.5 mg per kg b.wt. i.v.). Note that in these experiments thymidine kinase activity is expressed in per cent of activity measured in segments removed at time 0. Asterisks indicate statistical significance. Mean ± s.e.m.

The experiments with chronically denervated intestines described above suggested that capsaicin stimulates an axon reflex. In many organs calcitonin gene related polypeptide (CGRP) or substance P (SP) have been shown to be involved in axon reflexes [see e.g. references 20]–[22]. To elucidate if this also was the case in the present experiments two types of studies were performed. First, blocking the CGRP receptors (human 8–37 α-CGRP; 1 mg kg^−1^ b.wt. i.v.) or the SP receptors (more specifically the NK1 receptors: Sendide; 1 mg kg^−1^ b.wt. i.v.) abolished the increase of TK activity evoked by capsaicin (Figure 7; CGRP receptors: TK activity in capsaicin segments 78.5±9.7% of control segments; n = 6; p<0.001 compared to control experiments; NK1 receptors: TK activity in capsaicin segments 82.6±6.8% of TK activity in control intestines; n = 6; p<0.001 compared to control experiments) and the augmented incorporation of ^3^H-thymidine into DNA (Figure 8; CGRP receptors: incorporation in capsaicin segments 68.9±14% of control intestines; n = 6; p<0.01; NK1 receptors: incorporation in capsaicin segments 90.6±9.8% of control; n = 6; p<0.001) induced by luminal capsaicin. The doses used have been previously shown to block effects of CGRP and SP [22]–[24]. Second, infusing the neurotransmitters retrogradely in the carotid artery at rates of 10^−9^ mol per min for 1 h increased significantly intestinal TK activity compared to activity at the start of infusion (Figure 9; CGRP: TK activity 141.0±7.6% of activity at time 0; n = 5; p<0.05; SP: TK activity 143.9±20.7% of activity at time 0; n = 5; p<0.05).

There are two major types of cholinergic receptors, nicotinic and muscarinic. Above we described results from experiments with hexamethonium, a nicotinic receptor blocker. To block the muscarinic receptors, atropine (0.5 mg kg^−1^ b.wt.) was given i.v. Atropine significantly diminished the intestinal TK response to capsaicin (Figure 7; TK activity in capsaicin segments 91.8±5.6% of control; n = 6; p<0.001 compared to experiments without atropine) and the capsaicin evoked increase of ^3^H-thymidine incorporation into DNA (Figure 8; incorporation in capsaicin intestines 70.9±16.5% of control; n = 6; p = 0.002 compared to experiments without atropine).

In the atropine experiments it was possible to investigate the effect of atropine on rate of ^3^H-thymidine incorporation into DNA in control segments. After 2 h labeled nucleotide incorporation into DNA was 1017±81 DPM per mg DNA in animals not receiving atropine and 380±65 DPM per mg DNA in experiments with atropine (n = 6 in both groups; p<0.01 comparing the two groups). Hence, atropine lowered incorporation to about 40% of control suggesting an ongoing cholinergic, nervous influence in the absence of luminal capsaicin.

A possible interaction between muscarinic and peptidergic transmission was investigated by intravascular infusion of CGRP or SP after atropine administration. No CGRP or SP effect on TK activity could be demonstrated after giving atropine. (Figure 9; CGRP: 90.8±7.1% of TK activity at start of peptide infusion; n = 7; p<0.05 compared to CGRP experiments without atropine; SP: 73.8±5.7% of TK activity at time 0; n = 7; p<0.05). The simplest explanation for these observations is that CGRP and SP influence cholinergic neuron(s), which, in turn, control the intestinal stem cells.

**Figure 10:**
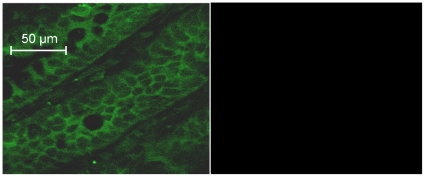
The localization of M5 muscarinic receptor immunoreactivity in intestinal crypts. The picture is photographed by confocal microscopy corresponding to a 4 µm thick section of the wall of the crypts. Hence, no crypt lumen is seen and the crypt cells are cross-sectioned. The left panel of the figure suggests that the M5 receptor is located to the plasma membrane of the crypt cells. Tissue sections exposed only to the secondary antibody did not exhibit any fluorescence (right panel).

To elucidate if muscarinic receptors were present on the intestinal stem/progenitor cells, we performed immunofluorescence studies using antibodies against the five different muscarinic receptors (M1–M5) described in the literature [25]. As on many other effector cells innervated by cholinergic neurons, immunoreactivity for more than one type of muscarinic receptors (M3 and M5) was identified in the intestinal crypts [25]. The left panel of figure 10 illustrates the distribution of the immunoreactivity for the M5 receptor in the crypts as revealed by confocal microscopy in a 4 µm thin section of a crypt wall. Hence, no crypt lumen is seen and the crypt cells are cross-sectioned. The disclosed fluorescence pattern is consistent with the immunoreactivy being located to cell membranes. The round structure in the middle of the figure is in all probability a cross-sectioned goblet cell surrounded by immunoreactivity. Tissue sections exposed only to the secondary antibody did not exhibit any fluorescence (Figure 10, right panel).

**Figure 11:**
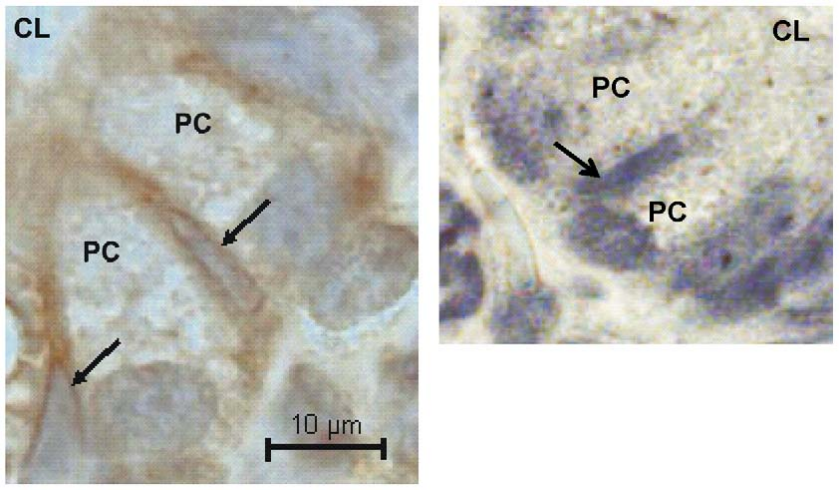
The localization of M3 muscarinic receptor immunoreactivity on intestinal stem cells. The stem cells, indicated by arrows, are slender cells located between the Paneth cells (PC) at the very base of the crypts [17], [26]. The left part of the figure indicates that the muscarinic receptor M3 is located to the plasma membrane of the presumed intestinal stem cells. The tissue section in the right panel was only exposed to the secondary antibody. Mayer's haematoxylin. CL  =  crypt lumen.

Cheng and Leblond [26] proposed that the so called base columnar cells were the intestinal stem cells generating all types of epithelial cells, a hypothesis recently supported by Barker *et al*. [17], [18]. Thus, the intestinal stem cells seem to be the slender cells situated between the Paneth cells. We studied the possible presence of muscarinic receptors on these cells. Two types of muscarinic receptors M3 and M5, were investigated. Immunoreactivity to M3 and M5 receptors was identified on the presumed stem cell membrane, as illustrated for the M3 receptor on the left panel of Figure 11. The right panel shows the appearance of the tissue when exposed only to the secondary antibody.

### Rate of epithelial renewal is decreased in capsaicin receptor knock-out mice

To investigate if the capsaicin receptor was of importance for cell renewal during “normal” conditions, experiments were performed on wild type mice and on mice lacking the TRPV1 gene [27]. ^3^H-thymidine (2 µCi) or BrdU (100 mg/kg b.wt.) were injected i.p. 2 h prior to extirpating the intestinal segments. ^3^H-thymidine incorporation into DNA was 529±46 DPM per mg DNA (n = 5) in control mice and 340±72 DPM per mg DNA (n = 5) in TRPV1 knock out mice (p<0.05).

The BrdU labeled cells were exclusively found in the intestinal crypts (*cf.* Figure 3). BrdU labeled cells in 215 crypts in wild type mice and 225 crypts in TRPV1^−/−^ mice were counted. The number of labeled cells per crypt was significantly lower in TRPV1^−/−^ than in wild type mice (right part of Figure 5; wild type: 11.4±0.2 BrdU labeled cells/crypt; knock-out: 9.2±0.2 BrdU labeled cells/crypt; n = 5 in both groups; p<0.01). Thus, in mice devoid of capsaicin receptors rate of ^3^H-thymidine incorporation into DNA and number of BrdU labeled crypt cells were significantly lower than in wild type mice.

## Discussion

The intestinal epithelium is the most rapidly self-renewing epithelium in adult mammals, being replaced every 3–5 day. Each crypt contains 4–6 long-lived stem cells that are constantly dividing. The daughter cells of the stem cells (here called progenitor cells) divide every 12–16 h as they move “upwards” along the crypts generating some 300 cells per crypt every day [28]. The present study provides evidence for a nervous control of intestinal stem/progenitor cells *in vivo*. We have demonstrated that luminal capsaicin (1.6 mM), stimulating thin nervous afferents, increases intestinal TK activity, ^3^H-thymidine incorporation into DNA and number of BrdU labeled crypt cells. No such effects were evoked by cholera toxin. Several observations strongly indicate that the observed capsaicin effects were nervously mediated. Hence, lidocaine, a local anesthetic, and antagonists to four different neurotransmitter receptors (muscarinic, nicotinic, NK1 and CGRP receptors) significantly attenuated the capsaicin response. A nervous involvement was also indicated by the failure of capsaicin to influence TK activity after allowing a degeneration of the extrinsic nerves (“chronic denervation”). Finally, the capsaicin effect was mediated by TRPV1 receptors, known to be located on extrinsic afferent nerves [20], the capsaicin effect being abolished by the TRPV1 receptor blocker capsazepine.

In the present study the measured TK activity has been taken as indicator of rate of cell renewal of the intestinal epithelium. The reasons for this are the following: TK is an important enzyme in DNA replication, phosphorylating the nucleoside before being incorporated into DNA. The enzyme exists in a cytosolic and mitochondrial form [15]. Cytosolic TK is present only in proliferating cells. Its importance for cell renewal is suggested by the observations that TK activity in the crypts is about 20 times higher than in the villi when expressed per unit tissue weight or DNA weight [29], [30]. This conclusion is also supported by our observations that ^3^H-thymidine or BrdU injected intravenously was localized almost exclusively to the nuclei of crypt cells (Figures 1, 2 and 3), as also shown by Lipkin [31] for labeled thymidine. Furthermore, the increased incorporation of thymidine into DNA and the increased number of BrdU labeled crypt cells in response to luminal capsaicin strongly suggest that the augmented TK activity reflected a change of cytosolic TK activity. Finally, nerve transmitter receptor antagonists concomitantly abolished the increase of TK activity and thymidine incorporation into DNA (compare Figures 7 and 8).

It might be argued that the increase of total intestinal TK activity reflects a proliferation of other cells than the stem/progenitor cells of intestinal crypts. Observations reported in the literature indicate that this is unlikely. Smooth muscle cells show almost no renewal in the mouse stomach [32]. Fibroblasts in the intestinal submucosa are renewed with turn over times varying between 100 and 130 days [33]. Hence, the two most dominating tissues of the intestinal wall, apart from the epithelium, show no proliferation within the short time of the present experiments.

The “chronic denervation” experiments are key experiments in the analysis of the anatomical arrangement of the nerves influencing intestinal stem/progenitor cells, since such experiments make it possible to elucidate if a studied reflex is confined to the intestinal wall (“intramural reflex”) or mediated via an axon reflex. An axon reflex may be influenced by severing an afferent neuron, whereas an intramural reflex will not. Thus, the periarterial nerves were cut and acute experiments were performed 2–3 weeks later, when the extrinsic innervation (afferent and efferent) had degenerated. The absence of extrinsic nerves was confirmed by immunohistochemical investigations of tyrosine hydroxylase, a rate limiting enzyme in catecholamine synthesis [19]. In chronically denervated rats luminal capsaicin failed to cause an increase of TK activity, indicating that capsaicin exerted its effect via an axon reflex. These observations are consistent with the TRPV1 receptors being present only on extrinsic afferent nerves of the gut [20].

There exist a number of criteria for neurotransmitter candidates to be accepted as true transmitters. Observations reported in this and other studies indicate that most of these criteria have been fulfilled with regard to SP and CGRP and the afferent nervous control of intestinal cell renewal. First, immunohistochemical investigations have revealed intestinal, extrinsic, afferent, nerves containing SP and CGRP [14], [20]. Second, subgroups of the capsaicin sensitive neurons show SP and/or CGRP immunoreactivity [20]. Third, blocking the receptors for NK1 and CGRP significantly attenuated the effect of luminal capsaicin on TK activity and ^3^H-thymidine incorporation (Figures 7 and 8). Fourth, intra-arterial administration of the two peptides significantly increased TK activity (Figure 9). Fifth, a release of SP or CGRP from stimulated afferent neurons has been reported in the literature [22], [34]–[36]. Thus, taken together the observations clearly suggest that SP and CGRP are involved in the capsaicin effect on epithelial cell renewal. The current results indicate, however, that the NK1 or CGRP receptors are not localized to the stem/progenitor cells, since atropine abolished the TK increase induced by intra-arterially infused SP or CGRP (Figure 9).

Capsaicin sensitivity is a trait of a subgroup of the unmyelinated C fibres but includes also some of the thinly myelinated Aδ fibres. Capsaicin stimulated receptors (TRPV1) are localized to extrinsic, afferent neurons usually considered to belong to the nociceptor system, a set of nerves that can be activated by stimuli (mechanical, thermal, chemical) capable of causing tissue damage. The TRPV1 receptor is located on free nerve endings of at least on two different types of neurons, one being a heat sensitive neuron and the other being a peptidergic, polymodal nociceptor neuron [37].

**Figure 12:**
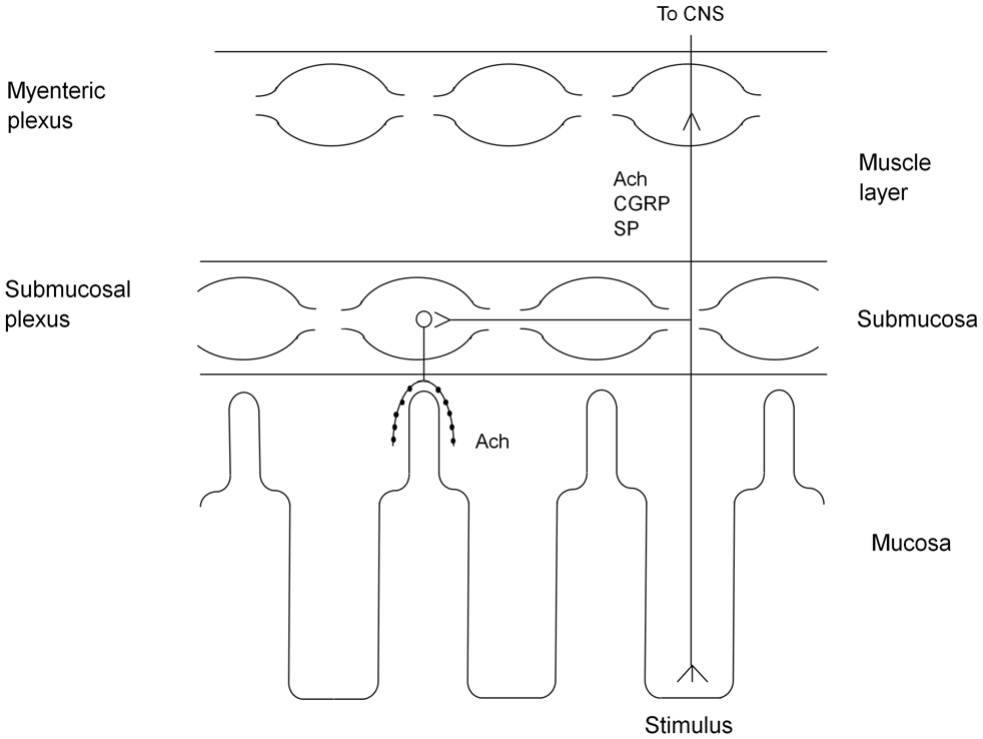
Model of axon reflex control of cell renewal in the small intestine. The model is in part based on the findings of the present study (*cf.* Table 1) and is consistent with known types of neurons in the intestinal wall. It implies that the direct control of the stem/progenitor cells is exerted by a cholinergic neuron, which, in turn, is under the influence of an axon reflex releasing CGRP/SP/acetylcholine. Ach: acetylcholine; CGRP: calcitonin gene related peptide; SP: substance P; CNS: central nervous system.

Combining observations reported in the literature with the findings of the current investigation it is possible to propose a model to explain the findings of the present study. The model is shown on Figure 12 and the observations that constitute the basis for the model are briefly summarized in Table 1. It should be underlined that Figure 12 represents the simplest model that can be constructed from our findings and the observations reported in the literature. It is a working hypothesis, which can be used when designing further experiments to prove or disprove the proposed model. The synapse between the afferent neuron and the cholinergic neuron has been located to the submucosal plexus in the figure for two reasons: first, most neurons controlling mucosal functions have their cell somas in the submucosal plexus. Second, luminal capsaicin transiently reduces CGRP-like immunoreactivity in the submucosal but not in the myenteric plexus [39].

**Table 1:** Observations supporting the model of Figure 12.

Capsaicin stimulates intestinal extrinsic but not intrinsic afferent nerves [14], [19]
A subset of capsaicin sensitive nerves releases SP and/or CGRP upon stimulation [14], [22], [38]
ChAT and CGRP immunoreactivity is co-localized in afferent, small diameter neurons [38]
The capsaicin effect on intestinal stem/progenitor cells is mediated by an axon reflex (TS)
Most axon reflexes in different organs mediate their effects by SP and/or CGRP [20], [21], [22]
The capsaicin effect on cell renewal is blocked by nicotinic, SP and CGRP antagonists (TS)
SP and CGRP stimulate intestinal cell renewal (TS)
The effect of SP and CGRP on cell renewal is mediated by cholinergic receptors (TS)
There are cholinergic receptors on intestinal stem/progenitor cells (TS)

TS: this study.

The proposed axon reflex control of intestinal cell renewal, may, however, not be the only nervous control of intestinal stem cells. We observed that atropine administration markedly attenuated nucleotide incorporation into DNA also in control segments. A rough calculation based on the averages of nucleotide incorporation (DPM per mg DNA) indicates that atropine decreased incorporation to about 40% of control. Hence, other enteric nerves than the thin extrinsic mucosal afferents may influence stem cell/progenitor cell proliferation, the cholinergic neuron being the final common pathway for more than one reflex. In fact, the results indicate that nervously released acetylcholine may play a very important role in the stem cell niche of the small intestine. With regard to the type of epithelial cells controlled by enteric nerves, observations reported by Bjerknes and Cheng [40], [41] indicate that the enteric nerves control the proliferation of the columnar daughter cells.

There is a fairly extensive literature dealing with muscarinic receptors and cell proliferation, in normal or cancerous cells (for reviews see [42], [43]). The muscarinic influence on proliferation is, at least in part, mediated by the “classical” signalling pathways for muscarinic receptors, the inositol-phospholipid signalling cascade [42] involving two second messengers, diacylglycerol and inositol 1,4,5-trisphosphate (IP_3_). This pathway is, however, not the only one mediating the muscarinic effect. Several studies report that the muscarinic receptor also is coupled to the mitogen-activated protein kinase (MAPK) pathway probably via a transactivation of the epidermal growth factor (EGF) [43]–[47] receptor located on the basolateral membrane of the crypt cell epithelium [48].

Besides luminal capsaicin we investigated the effect of cholera toxin on TK activity and thymidine incorporation into DNA. It was observed that the toxin, if anything, decreased cell renewal to judge by attenuated nucleotide incorporation into DNA. This is consistent with earlier observations that the final neurotransmitter in the secretory reflex activated by cholera toxin is not acetylcholine but vasoactive intestinal polypeptide (VIP) [49]–[52]. Atropine does not attenuate cholera secretion [13] excluding a muscarinic mechanism. Furthermore, in contrast to the present findings chronic denervation does not influence the secretory response to cholera toxin [53]. We conclude that cholera toxin and capsaicin stimulate two different reflexes with different final neurotransmitters, one being confined to the intestinal wall (cholera toxin reflex), releasing VIP, and the other being an axon reflex (capsaicin reflex) releasing acetylcholine at the effector cell.

We have earlier reported that ENS is of great importance in explaining fluid secretion in acute diarrhoea [11]–[13]. As pointed out in the introduction diarrhoea can be looked upon as being an innate immunity response, since the secreted fluid dilutes the noxious agent(s). Furthermore, fluid secretion is often accompanied by a nervously induced motility response propelling the intestinal contents aborally [54]. Capsaicin activated intestinal nerves evoke an intestinal vasodilatation [22] and may be involved in the control of mucus secretion [55] and intestinal homing of leukocytes [56]. Taken together with the present observations it suggests that ENS coordinates several innate immunity mechanisms in the gut.

As discussed above the capsaicin receptor TPRV1 is usually considered to be a nociceptor sensing potentially noxious stimuli. It may, however, also participate in physiological mechanisms. The contents of the intestinal lumen influences mucosal morphology, parenteral feeding leading to a decreased mucosal thickness [57]. This is usually explained in terms of trophic factors, such as hormones. However, the studied reflex may be involved. There are at least three physiological ways by which the intestinal contents may influence mucosal afferent nerves. First, the TRPV1 receptor seems to be an osmoreceptor activated by hyperosmolality [58]. A tissue hyperosmolality has been demonstrated in the upper parts of intestinal villi in several species with different techniques [59], measuring about 800 mOsm in humans and cats provided the intestinal contents contain sodium and glucose. We propose that TRPV1 receptors are activated by a villous tissue hyperosmolality. Such a mechanism may explain the present findings in the knock-out mice implying that the capsaicin receptor is important for cell renewal even in the absence of luminal capsaicin.

Second, intake of food leads to the presence of pancreatic proteases in the intestinal lumen. It has been shown that proteinase-activated receptors 1 and 2 (PAR-1, PAR-2) are present on the apical border of enterocytes and activated by trypsin [60], [61]. In the case of PAR-2 receptors indirect evidence supports the proposal that a stimulation of this receptor may influence firing in afferent nerves from the gut. Thus, Cenac *et al*. [62], [63] and Nguyen *et al*. [64] reported that exposing the colonic mucosa to a PAR-2 receptor agonist causes a neurogenic inflammation via capsaicin sensitive nerves. Furthermore, the PAR-2 induced inflammation was attenuated by NK1 and CGRP antagonists.

Third, one may speculate that the intestinal contents via the discharge of hormones from enteroendocrine cells activate mucosal afferents to increase epithelial cell renewal. A similar chain of events has been proposed for the activation of intramural secretory nervous reflexes by e.g. cholera toxin [11], [13] and rotavirus [12]. The finding reported by Bjerknes and Cheng [65] that glucagon-like-peptide-2 exerts its stimulatory effect on epithelial proliferation via nerves may be explained by such a mechanism.

Microorganisms in the intestinal lumen are obvious examples of potential noxious agents. Their presence may be recorded by the intestinal epithelium, functioning as a mucosal defence early warning system. Using membrane-associated or intracellular pattern-recognition molecules, such as toll-like receptors and Nod proteins, the epithelial cells sense the microorganisms [66]. This, in turn, may evoke a release of cytokines, prostaglandins and/or nitrous oxide from the enterocytes [67], biologically potent compounds with receptors on capsaicin sensitive nervous dendrites. Invading microorganisms may evoke an inflammatory response. Many of the substances participating in an inflammation, such as cytokines, prostaglandins, adenosine, bradykinin, histamine and 5-hydroxytryptamine, may directly or indirectly via activation of immunocytes, stimulate the studied axon reflex [38], [68], [69]. A morphological characteristic of ulcerative colitis is the “reactive“ hyperplasia of the epithelium [70]. One may speculate this hyperplasia may, at least in part, reflect an inflammatory activation of the axon reflex controlling intestinal stem/progenitor cells. Similar mechanisms may exist in connection with inflammation of other mucosae, as in the air ways and the urinary tract.

It is well established that there is a relationship between tissue repair and cancer, implying that chronic tissue injury may lead to malignancy (see e.g. [71]). A well-known example is colon cancer developing in longstanding ulcerative colitis. It has been suggested that overall risk of cancer development depends on the number of activated stem cells. Any repeated stimulus leading to a chronic increase in stem cell numbers could result in a higher overall frequency of cancer formation [28], [72]. Furthermore, it has been pointed out that the efficiency of carcinogenesis in tumour models is increased when combining mutagenic agents with agents that induce cell proliferation [73]. In this context it is interesting to note that Frucht and coworkers [74] found that 6 out of 10 human colon cancer cell lines were provided with functional muscarinic cholinergic receptors. Therefore, in view of the findings of the present study, one may pose the question if long standing activation of mucosal afferent nerve fibres by e.g. inflammation, may, via a cholinergic stimulation of muscarinic receptors and transactivation of EGF receptors, contribute to the development of cancer by their influence on epithelial proliferation.

## Methods

### Ethical approval

The protocol used in the present experiments was approved by the Ethical committee for animal experimentation at Gothenburg University, Gothenburg, Sweden (approval 292-2009). The experiments were performed in accordance with the recommendations issued by the Swedish Department of Agriculture.

### Rat experiments

Experiments were performed on male Sprague-Dawley rats, weighing 240–450 g (Möllegaards Breeding Centre Ltd, Ejby, Denmark or B&K Universal AB, Sollentuna, Sweden). The rats were housed for at least 7 days prior to experiments in animal quarters (22°C, 60% relative humidity, artificial lighting between 06.00 and 18.00 h). Before the experiments the rats were deprived of food for at least 12 h. All experiments were carried out between 9.30 and 15.00.

#### Anesthesia and operative procedures

Anesthesia was induced with pentobarbital (60 mg/kg body wt.) i.p. in all experiments except those in which the intestinal segments were chronically denervated (see below). A tracheal cannula was inserted and a femoral vein was cannulated. Arterial pressure was continuously monitored in a femoral artery on a Grass polygraph. The arterial catheter was also used to maintain anesthesia by a continuous infusion of α-chloralose (Alfa Aesar GmbH & Co, Karlsruhe, Germany; 3 mg/ml; 20 µL/min) in a solution containing 138 mM glucose and 33 mM NaHCO_3_. Body temperature was kept around 38°C with a heated operating table. At the end of the experiments the animals were killed by an i.v. injection of a saturated KCl solution while still anesthetized.

A midline abdominal incision was performed. The influence of the extrinsic autonomic nervous supply to the intestinal segments was minimized by cutting the nerves along the superior mesenteric artery.

#### Experimental procedures

Two to six 2–6 cm long jejunal segments were isolated 5–10 cm below the ligament of Treitz. The experiments with capsaicin were performed using two types of preparations. In most experiments only the proximal ends of the segments were cannulated with plastic tubing and the intestinal segment was perfused by means of a pump at a rate of 20 µL per min (“perfusion experiments”). In other experiments the distal ends were also provided with plastic tubing closed by plugs (“non-perfusion experiments”). The original capsaicin solution contained 16 mM capsaicin dissolved in a solution containing Tween 80 (1 part), ethanol (1 part) and NaCl (0.9%; 8 parts). The concentration of capsaicin most often used (1.6 mM) was obtained by a 10 times dilution of the 16 mM solution with physiological saline. The control solution did not contain capsaicin. In some experiments the intestinal mucosa was exposed to cholera toxin (List Biological Laboratories, Campbell, California; 20 µg per mL physiological saline) or to saline. The TK experiments (see below) lasted for 60 min, whereas the [methyl-^3^H]thymidine and BrdU experiments were prolonged to 120 min.

At predetermined times intestinal segments were removed. Macroscopically apparent Peyer's patches were removed. Intestinal segments to be utilized for immunohistochemistry or autoradiography were placed in buffered a 4% paraformaldehyde (pH 7.0) solution. Intestinal segments to be used for estimation of thymidine kinase or DNA were immediately frozen in liquid nitrogen and kept at −20°C or −85°C until determination. In all types of experiments the choice of segments was randomized.

#### Lidocaine experiments

In one type of experiments the intestinal mucosa was exposed to lidocaine, a local anaesthetic, together with capsaicin. About 10 min before exposing the intestinal segment to capsaicin the segment was flushed with a 1% lidocaine (w/v) in physiological saline. Furthermore, the capsaicin solution contained 0.2% lidocaine (perfusion experiments) or 1% lidocaine (non-perfusion experiments). Control solutions lacked capsaicin. One may argue that tetrodotoxin, a more specific nerve blocking agent than lidocaine, would have been more appropriate. However, the use of tetrodotoxin in *in vivo* experiments is greatly complicated by its high general toxicity for the experimental animal.

#### Neurotransmitter receptor blockade

Several neurotransmitter antagonists were tested. Atropine (a muscarinic receptor blocker; 0.5 mg kg^−1^ b.wt.) or 8-37 α-GGRP (a CGRP receptor blocker; 1 mg kg^−1^ b.wt;) was administered i.v. at times 0 and 60 min. Hexamethonium (a nicotinic receptor blocker; 10 mg kg^−1^ b.wt) or Sendide (1 mg kg^−1^ b.wt; a NK1 receptor blocker) was administered i.v. every 30. min.

#### Neurotransmitter i.a. infusions

SP or CGRP were infused i.a. retrogradely via a catheter in the left carotid artery at a rate of 10^−9^ mol per min. The peptides were dissolved in a 1% albumin-saline solution. In the cholera toxin and neurotransmitter experiments the intestines were not perfused.

#### BrdU experiments

In 5 experiments bromodeoxyuridine (BrdU; a thymidine analogue; Roche Diagnostics Scandinavia, Bromma, Sweden; dissolved in physiological saline; 50 mg/kg b.wt.) was administered i.v.

#### Chronic denervation experiments

In 5 experiments the intestinal segments were denervated periarterially 2–3 weeks prior to the acute experiments. The rats were anesthetized with ketamin (Ketalar, Pfizer Animal Health; 75 mg/kg b.wt. i.p.) and medetomidine (Domitor vet., Orion Oyj, Espoo, Finland; α_2_-adrenoreceptor agonist; 0.5 mg/kg b.wt. i.p.). The superior mesenteric artery was dissected free about 10 mm from its origin from the aorta. A wide portion of the tissue surrounding the artery and containing the periarterial nerves was divided between ligatures. To ablate any nerve fiber bundles that were not severed by this procedure the arterial vessel was painted with a 5% phenol solution. The abdomen was closed by ligatures and the animals were placed in a heated cage. The animals were aroused by giving atipamezole (Antisedan vet., Orion Oyj, Espoo, Finland; α_2_-adrenoreceptor antagonist; 0.5 mg/kg b.wt. i.p.). The acute experiments carried out later were performed as described above (“perfusion experiments”). The success of denervation was tested by an immunohistochemical investigation of the presence of thyrosine hydroxylase (marker of adrenergic nerve fibers; see below).

#### Determination of thymidine kinase activity

Thymidine kinase (TK) catalyses the phosphorylation of deoxythymidine to deoxythymidine-5-phosphate. TK activity in the intestine was measured in duplicate samples as described [30], [75], [76]. Intestinal biopsies were homogenized (Polytron PT 1200 homogeniser) in a 2 ml Eppendorf tube with a Tris-phosphate buffer (pH 7.3) at 0–4°C and centrifuged at 16 000× g for 20 min, a g-force above that which enriches the pellet with mitochondria [30]. Supernatant (100 µl) was added to a reaction mixture (volume 1.275 mL) containing ATP (12 mM), phosphoglycerate (10 mM), MgCl_2_ (4 mM), NaF (3 mM), thymidine (0.4 mM) and [methyl-^3^H]-thymidine (12.5 or 25 µCi corresponding to 0.2 or 0.4 µM; Amersham Biosciences, Buckinghamshire, U.K.) in a 0.05 M TrisHCl buffer (pH 8). After a 20 min incubation period at 37°C in a metabolic shaker, the reaction was stopped by filtering the solution with a small volume of NaCOOH through an ion exchange filter (Whatman DE 81) This filter retained labelled dTMP, whereas free thymidine was washed away. After repeated washings with NaCOOH and water, the filter was placed in a scintillation counting vial together with 1 ml of a solution containing HCl (2 M) and NaCl (1 M) in a 1∶1 relationship. After 10 min scintillation fluid (Quickszint 361, Zinsser Analytical, Frankfurt, Germany) was added. The radioactivity was measured in a Packard 1900 TR Liquid scintillation analyser.

#### Thymidine incorporation into DNA

[Methyl-^3^H]thymidine (25 or 50 µCi; Amersham Biosciences) was given i.v. half an hour after exposing an intestinal segments to cholera toxin or just prior to perfusing intestinal segments with 1.6 mM capsaicin or a control solution. DNA was isolated as described [77]. The amount of DNA was estimated from measurements of absorbance at 260 nm [78]. The radioactivity was measured in a Packard 1900 TR Liquid scintillation analyser.

#### Immunohistochemistry

Biopsies from the rat small intestine immersed in buffered 4% paraformaldehyde (pH 7.0) were subsequently embedded in paraffin.

Bromodeoxyuridine (BrdU) is a thymidine analogue, which can be localized in tissue with histochemical techniques. It is incorporated into the DNA during the S phase making it possible to quantify the number of newly formed cells. In the present experiments intestinal segments were extirpated 2 h after giving BrdU (50 mg/kg b.wt.) i.v. The tissue was immersed in buffered 4% paraformaldehyde (pH 7.0), embedded in paraffin and cut at 4 µm. Endogenous peroxidase was inactivated with 3% H_2_O_2_. Non-specific protein binding was blocked with 4% normal donkey or 1.5% normal rabbit serum in Tris-buffered saline (TBS) for 30 min. The sections were exposed to monoclonal rat BrdU antibodies (0.5 µg/ml; Adb Serotec, Oxford, UK) over night at 4°C followed by incubation with avidin-biotin-peroxidase complex (ABC Elite, Vector laboratories, Inc., Burlingame, California) for 60 min at room temperature. The immunoreactivity was visualized with 3.3′-diaminobenzidine tetrahydrochloride (DAB; 0.50 mg/ml). Most biopsies were counterstained with Mayer's hematoxyline. As a negative tissue sections were immunostained without exposure to the primary antibody, which did not result in any staining of the tissue. The number of BrdU labelled crypt cells was counted in coded tissue sections at 200 times magnification using an Olympus BX60 microscope. The crypts had to fulfil two criteria to be included when estimating number of BrdU labelled cells. First, a crypt lumen was seen along the length of the crypt. Second, the bottom of the crypt was located close to the submucous layer.

Immunohistochemical investigation of muscarinic receptor (mAChR) expression was in some experiments undertaken on 20 µm thick sections, which were deparaffined and re-hydrated by heating the slides to 60°C for one hour followed by subjection to xylene, decreasing concentrations of ethanol and TBS. The sections were then immersed in a 10 mM citrate buffer (pH 6.0) and microwaved for four cycles of 6 min. Endogenous peroxidase was inactivated with 0.03% H_2_O_2_. Non-specific protein binding was minimized with 5% bovine serum albumin (BSA) in TBS for 30 min. The sections were then incubated overnight at room temperature in a humidified chamber with polyclonal rabbit anti-mAChR subtype specific antibodies (Research and Diagnostic Antibodies, Berkley, California) diluted 100 times in TBS containing 1% BSA. The presence of the muscarinic receptor immunoreactivity was revealed using Alexa Fluor 488 goat anti-rabbit Ig G (Molecular Probes, Eugene, Oregon) and analyzed using a Radiance 2000 Confocal Imaging System (Bio-Rad, Hercules, California) and the LaserSharp 2000 software (Bio-Rad).

Certain other sections (4 µm thick) were treated with an immunoenzymatic method. Generally speaking, the method was similar to that described above with two modifications: endogenous peroxidase was inactivated with 0.6% H_2_O_2_ and the sections were stained with a secondary biotinylated goat antirabbit antibody (Dako, Sweden AB, Solna, Sweden) followed by a horseradish peroxidase-labeled avidin-biotin complex (Dako). The colour was then developed with AEC substrate chromogen (Dako). The sections were analyzed with an Olympus BX60 microscope. Control sections were immunostained without exposure to the primary antibody. It has previously been shown that the binding of the muscarinic receptor antibodies used in the present study are blocked by preincubation with its specific antigen [79].

We have used the adrenergic innervation to test presence or absence of external innervation after severing the intestinal periarterial nerves. This method is based on the absence of adrenergic nerves in the ENS that are confined to the intestinal wall. Therefore, adrenergic nerves are extrinsic and their absence in intestinal segments indicates a successful surgical denervation. This method has been used extensively [for experiments on rats], [ see references 80]–[82]. Tissue from every experimental segment was immersed in buffered 4% paraformaldehyde (pH 7.0) and embedded in paraffin. Four µm thick tissue sections were collected at a regular distance of 40 µm between biopsies. The sections were deparaffined in xylene and decreasing concentrations of ethanol and then immersed in 10 mM citrate buffer (pH 6.0) at 100°C. Endogenous peroxidise activity was blocked by 0.3% H_2_O_2_ The sections were exposed to a mouse, monoclonal, tyrosine hydroxylase antibody (Affinity BioReagents, AH Diagnostics, Skärholmen, Sweden) for 45 min at room temperature. A biotin rat anti-mouse IgG1 (BD Biosciences Sweden, Stockholm, Sweden) was used as a secondary antibody. After exposure to a peroxidase-conjugated avidine-biotin complex (ABC-complex, Dako) the sections were stained with an AEC-substrate (Dako) and counterstained with Mayer's hematoxyline. Control sections were immunostained without exposure to the primary antibody, which did not result in any staining of the tissue.

#### Autoradiography experiments

Methyl-^3^H-thymidine (50 or 100 µCi; specific activity 70–75 Ci/mmol; Amersham Biosciences, UK) in physiological saline, was given i.v. four or six hours prior to extirpation of the intestinal segment. After embedding in paraffin, the biopsy was cut at 4 µm. For autoradiography the histological sections were deparaffinated with xylene and dehydrated with ascending concentrations of ethanol. The sections were dipped in a photographic solution (Ilford K2) for 10 s, exposed usually for about 4 weeks, developed and fixed. The sections were then stained (Van Gieson). The autoradiographs were analyzed by conventional bright field microscopy as well as by dark field microscopy.

### Mouse experiments

Experiments were performed on mice devoid of the capsaicin receptor (TRPV1 [27]; strain B6.129X1-Trpv1) purchased from The Jackson Laboratory (Bar Harbor, Maine). Strain B6129SF2/J was used as controls as recommended by the distributor. The mice were kept in animal quarters under standardized environmental conditions (see above). At the time of the experiments the animals were 10 weeks old.

^3^H-thymidin (2 µCi) or BrdU (100 mg/kg body weight) dissolved in physiological saline was administered i.p.. Two h later the mice were killed with an overdose of sodium pentobarbital i.p. ^3^H-thymidine incorporation into DNA was determined and BrdU labeled cells were stained and counted as described for rat experiments.

### Chemicals

Peptides were bought from Bachem AG, (Bubendorf, Switzerland). All other chemicals, for which any supplier has not been indicated in text, were purchased from Sigma-Aldrich Sweden, Stockholm, Sweden.

### Recordings of heart rate

Heart rate was monitored on line with a rate meter coupled to the blood pressure recorder sensing the arterial pressure oscillations and recorded on a Grass polygraph.

### Statistics

Non-parametric statistics were used throughout. Significance between paired observations was determined using Wilcoxon matched-pairs test. The Mann-Whitney U test was utilized when comparing independent groups. All calculations were performed with SPSS for Windows. Level of statistical significance was set at p<0.05.
